# *Alicyclobacillus* spp.: New Insights on Ecology and Preserving Food Quality through New Approaches

**DOI:** 10.3390/microorganisms3040625

**Published:** 2015-10-10

**Authors:** Emanuela Ciuffreda, Antonio Bevilacqua, Milena Sinigaglia, Maria Rosaria Corbo

**Affiliations:** Department of the Science of Agriculture, Food and Environment, University of Foggia, Via Napoli 15, 71122 Foggia, Italy; E-Mails: emanuela.ciuffreda@unifg.it (E.C.); antonio.bevilacqua@unifg.it (A.B.); milena.sinigaglia@unifg.it (M.S.)

**Keywords:** isolation, source, genotyping, phenotyping

## Abstract

*Alicyclobacillus* spp. includes spore-forming and thermo-acidophilic microorganisms, usually recovered from soil, acidic drinks, orchards and equipment from juice producers. The description of the genus is generally based on the presence of ω-fatty acids in the membrane, although some newly described species do not possess them. The genus includes different species and sub-species, but *A. acidoterrestris* is generally regarded as the most important spoiler for acidic drinks and juices. The main goal of this review is a focus on the ecology of the genus, mainly on the species *A. acidoterrestris*, with a special emphasis on the different phenotypic properties and genetic traits, along with the correlation among them and with the primary source of isolation. Finally, the last section of the review reports on some alternative approaches to heat treatments (natural compounds and other chemical treatments) to control and/or reduce the contamination of food by *Alicyclobacillus*.

## 1. Introduction: The General Traits of *Alicyclobacillus* spp.

The genus *Alicyclobacillus* belongs to the family of *Alicyclobacillaceae* [1], and consists of a group of thermo-acidophilic, strictly aerobic, heterotrophic, and spore-forming bacteria [2,3].

First, alicyclobacilli were placed in the genus *Bacillus*, as they share with bacilli the characteristic of endospore formation. However, phylogenetic analysis based on sequence comparisons of the 16S rRNA showed that the species of the genus *Alicyclobacillus* belonged to a distinct line of descent within the low G + C (guanine + cytosine) Gram-positive lineage of *Bacteria* also including the closely related facultatively autotrophic species of *Sulfobacillus* spp. [2,4,5]. Therefore, in 1992 they were allocated to a new genus called *Alicyclobacillus* due to the presence of ω-cyclohexyl or ω-cycloheptyl fatty acids as the major natural membrane lipid component [6,7]. These ω-alicyclic fatty acids may be associated with the heat and acid resistance of *Alicyclobacillus* spp. [8], as they are responsible for the ability to survive typical pasteurization regimes applied during juice manufacturing, since *Alicyclobacillus* spp. may be present on fruit surfaces contaminated by soil during production and harvesting [9]. In single-strength juice, these microorganisms find a favourable environment for germination, growth, and spoilage [8].

The genus originally consisted of three species, *A. acidocaldarius*, *A. acidoterrestris* and *A. cycloheptanicus*. Nowdays, it consists of 22 species isolated from various habits (Table 1).

**Table 1:** Phenotypic Characteristics of *Alicyclobacillus* spp.

Species	Source of Isolation	Temp. Range (°C)	Optimum Temperature (°C)	pH Range	Optimum pH	ω-Cyclohexane/ω-Cicloheptane Fatty Acids	References
*A. acidiphilus*	acidic beverages	20–55	50	2.5–5.5	3.0	ω-cyclohexane	[10]
*A. acidocaldarius*	soil, fruits, syrup	35–70	55–60	2.5–6.0	4.5	ω-cyclohexane	[2]
*A. acidoterrestris*	soil, acidic beverages	20–55	40–50	2.0–6.0	3.5–4.5	ω-cyclohexane	[2]
*A. aeris*	copper mine	25–35	30	2.0–6.0	3.5	none	[11]
*A. cellulosilyticus*	cedar chips	40.0–67.5	55	3.5–6.5	4.8	ω-cyclohexane	[12]
*A. contaminans*	juices	35–60	50–55	3.0–6.0	4.0–4.5	none	[13]
*A. cycloheptanicus*	soil	30–55	50	3.0–5.5	4.0	ω-cycloheptane	[2]
*A. dauci*	spoiled mixed juice	20–50	40	3.0–6.0	4.0	ω-cyclohexane	[14]
*A. disulfidooxidans*	wastewater sludge	04–40	35	0.5–6.0	1.5–2.5	ω-cyclohexane	[15]
*A. fastidiosus*	soil, beverages	20–55	40–45	2.0–5.5	4.0–4.5	ω-cyclohexane	[13]
*A. ferrooxydans*	solfataric soil	17–40	28	2.0–6.0	3.0	none	[16]
*A. herbarius*	herbal tea	35–65	55–60	3.5–6.0	4.5–5.0	ω-cycloheptane	[17]
*A. hesperidum*	solfataric soil	35–60	50–53	2.5–5.5	3.5–4.0	ω-cyclohexane	[18]
*A. kakegawensis*	soil	40–60	50–55	3.0–6.5	4.0–4.5	ω-cycloheptane	[13]
*A. macrosporangiidus*	beverages, environments	35–60	50–55	3.0–6.5	4.0–4.5	none	[13]
*A. pomorum*	fruits	30–60	45–50	2.5–6.5	4.5–5.0	none	[19]
*A. sacchari*	sugar	30–55	45–50	2.0–6.0	4.0–4.5	ω-cyclohexane	[13]
*A. sendaiensis*	soil	40–65	55	2.5–6.5	5.5	ω-cyclohexane	[20]
*A. shizuokaensis*	soil	35–60	45–50	3.0–6.5	4.0–4.5	ω-cycloheptane	[13]
*A. tengchongensis*	hot spring soil	30–50	45	2.0–6.0	3.2	ω-cycloheptane	[21]
*A. tolerans*	solfataric soil	20–55	37–42	1.5–5.0	2.5–2.7	ω-cyclohexane	[15]
*A. vulcanalis*	geothermal pool	35–65	55	2.0–6.0	4.0	ω-cyclohexane	[22]

Twelve species of *Alicyclobacillus*, namely *A. acidocaldarius*, *A. acidoterrestris* [2], *A. hesperidum* [18], *A. acidiphilus* [10], *A. sendaiensis* [20], *A. disulfidooxidans, A. tolerans* [15], *A. fastidiosus*, *A. sacchari* [13], *A. vulcanis* [22], *A. cellulosilyticus* [12], and *A. dauci* [14], contain ω-cyclohexane fatty acids, whereas *A. cycloheptanicus* [23], *A. kakegawensis*, *A. shizoukensis* [13], *A. herbarius* [17], and *A. tengchongensis* [20] contain ω-cycloheptane fatty acids.

Recent studies [24] pinpointed the lack of these fatty acids in *A. aeris* [20], *A. ferrooxydans* [16], *A. pomorum* [19], *A. macrosporangiidus* and *A. contaminans* [14]. These species also possess a lot of phenotypic characteristics different from the classical traits of *Alicyclobacillus* spp., including growth temperature, assimilation of various carbon sources, production of acids from a range of compounds, and the ability to grow chemoautotrophically using ferrous iron, elemental sulphur and tetrathionate as electron donors [11,13,19]. However, genotypic analysis showed that they were phylogenetically related to members of the genus *Alicyclobacillus* [11,13,16,19].

Generally, interest in *Alicyclobacillus* spp. focused on the study of the role of ω-cyclic fatty acids and hopanoids on membrane function [25,26]. These ω-alicyclic fatty acids could be associated with the strong heat and acid resistance of *Alicyclobacillus* spp. [8]. Kanneberg *et al.* [26] demonstrated that lipids, which contain ω-cyclohexane fatty acid, packed densely, resulting in low diffusion at high temperatures. Wisotzkey *et al.* [2] proposed that this property provided an advantage at high temperatures or low pH. Lipids containing fatty acids with a cyclohexane ring could stabilize the membrane structure and maintain the barrier functions of prokaryotic membranes at high temperatures [26]. These fatty acids might contribute to the heat resistance of *Alicyclobacillus* by forming a protective coating with strong hydrophobic bonds. These hydrophobic bonds might stabilize and reduce membrane permeability in extreme acidic and-high temperature environments [2,26,27].

However, some authors reported that the presence of ω-cyclohexyl fatty acids is not essential in protecting alicyclobacilli from high temperatures and low pH, because there are other microorganisms, such as *Curtobacterium pusillum* [28] and *Propionibacterium cyclohexanicum* [29], that also possess ω-alicyclic acids, and are neither thermophilic nor acidophilic (*Propionibacterium cyclohexanicum* is an acidotolerant bacterium).

The possible way to justify the adaptation to extreme environments of alicyclobacilli might be the presence of hopanoids in their cells [7,8,25]. The hopane glycolipids are structurally similar to cholesterol and have a condensing effect on the cell membrane due to a decrease of the acyl chain lipids’ mobility. At low pH the condensing action hinders the passive diffusion of protons through the membrane, facilitating the establishment of an approximately neutral cytoplasmic pH [25]. The low membrane viscosity induced by branched-chain fatty acids is thus counterbalanced by the presence of a higher concentration of hopanoids, leading to a more stable membrane.

## 2. Characteristic of *Alicyclobacillus* spp.

The genus *Alicyclobacillus* is most closely related to the genus *Bacillus* and comprises thermophilic-acidophilic spore-forming bacteria. The genome study of this organism was fundamental for its phylogenetic position. In GenBank there are the complete genome sequences of each species. 16S ribosomal RNA (rRNA) sequencing is a common amplicon sequencing method used to identify and compare bacteria present within a given sample. 16S rRNA gene sequencing is a well-established method for studying phylogeny and taxonomy of samples from complex microbiomes or environments that are difficult or impossible to study.

Wisotzkey *et al.* [2] proposed that 16S rRNA gene sequences must be at least 92% similar to belong to the genus *Alicyclobacillus*. Within closely related species, especially belonging to the *A. acidocaldarius* group, the similarity is over 98%. Table 2 reports the similarity level of 16S rRNA and G + C content of DNA in *Alicyclobacillus* spp.

**Table 2:** Genotypic characteristics of *Alicyclobacillus* spp.

Species	DNA G + C Content (%)	Homology with 16S rRNA of Some Other Species of the Genus	References
*A. acidiphilus*	54.1	*A. acidoterrestris* (96.6%)	[10]
*A. acidocaldarius*	61.89	*A. acidoterrestris* (98.8%)	[30]
*A. acidoterrestris*	51.5	*A. acidocaldarius* (98.8%)	[2]
*A. aeris*	51.2	*A. ferrooxydans* (94.2%)	[11]
*A. cellulosilyticus*	60.8	*A. macrosporangiidus* (91.9%)	[12]
*A. contaminans*	61.1–61.6	*Alicyclobacillus* (92.3%–94.6%)	[13]
*A. cycloheptanicus*	57.2	*Alicyclobacillus* (92.7%–93.2%)	[2]
*A. dauci*	49.6	*A. acidoterrestris* (97.4%) and *A. fastidiosus* (97.3%)	[14]
*A. disulfidooxidans*	53	*A. tolerans* (92.6%)	[15]
*A. fastidiosus*	53.9	*Alicyclobacillus* (92.3%–94.6%)	[13]
*A. ferrooxydans*	48.6	*A. pomorum* (94.8%)	[16]
*A. herbarius*	56.2	*Alicyclobacillus* (91.3%–92.6%) and *Sulfobacillus thermosulfidooxidans* (84.7%)	[17]
*A. hesperidum*	60.3	*Alicyclobacillus* (97.7%–98%)	[18]
*A. kakegawensis*	61.3–61.7	*Alicyclobacillus* (92.3%–94.6%)	[13]
*A. macrosporangiidus*	62.5	*Alicyclobacillus* (92.3%–94.6%)	[13]
*A. pomorum*	53.1	*Alicyclobacillus* (92.5%–95.5%)	[19]
*A. sacchari*	56.6	*Alicyclobacillus* (92.3%–94.6%)	[13]
*A. sendaiensis*	62.3	*A. vulcanis* (96.9%)	[22]
*A. shizuokaensis*	60.5	*Alicyclobacillus* (92.3%–94.6%)	[13]
*A. tengchongensis*	53.7	*Alicyclobacillus* (90.3%–92.8%)	[21]
*A. tolerans*	48.7	*Alicyclobacillus* (92.1%–94.6%) and *S. thermosulfidooxidans* (87.7%)	[15]
*A. vulcanalis*	62	*A. acidocaldarius* (97.8%)	[22]

G + C content in DNA is 48.6% to 63.0%; it is *ca*. 62% for *A. acidocaldarius*, and 55% for the other species of *Alicyclobacillus* [13,31]. The content of G+C in *A. acidoterrestris* varies between 51.5% and 53.3% depending on the strain, with the type strain, *A. acidoterrestris* DSM 3922^T^, having a G + C amount of 51.5% [20,31,32].

Guaiacol production is a common trait of the genus, although the amount of this compound is greatly variable [32].

Regarding sugar metabolism, there is acid but not gas production. Different species could be divided into different phenotypic groups, depending on sugar metabolism, although there is considerable variation within species [8,13]. As in *Bacillus*, the major respiratory lipoquinone of *Alicyclobacillus* is menaquinone-7 (MK-7) [13]. The branched respiratory chain of thermophilic bacilli is quite complex; MK-7 plays a fundamental role, as it is reduced by several dehydrogenases (malate, succinate, NADH). NADH dehydrogenase is of type II and does not translocate H^+^. Energy conservation occurs upon menaquinol oxidation by b_6_c_1_ complex and Cyt *caa3*. However, at high temperatures the concentration of dissolved oxygen quickly decreases, thus the Cyt pattern of cells suddenly changes [33].

Concerning the other metabolic traits, starch and gelatin hydrolysis, catalase and oxidase tests are generally species- and strain-dependent, as well resistance to 5% NaCl, nitrate reduction, catalase and oxidase activities [34].

*Alicyclobacillus* spp. were described as strictly aerobic microorganisms; however, some authors reported alicyclobacilli growth with 0.1% oxygen in the headspace [13]. Alicyclobacilli generally grow as planktonic and free cells, but they could also produce a biofilm under favorable conditions [34,35].

*Alicyclobacillus* spp. are the type organisms to study and characterize thermostable and non-conventional enzymes (endoglucanase, esterases, α-galactosidase, arabinose isomerase, amylase and many others) [36,37,38,39,40]. These enzymes represent unique compounds due to their resistance to extreme conditions, as well as to their peculiarities in terms of structure [41], e.g.:

1. Lower number of charged residues. The α-amylases extracted from *Alicyclobacillus* spp. contain *ca*. 30% fewer charged residues than their closest relatives.
2. Acidic and basic residues. More basic residues are exposed on the surface, whereas the acidic groups are buried on the interior.
3. Salt bridges. Pechkova *et al.* [42] reported that an increase number of salt bridges results in greater compactness of the structure and thereby contributes to thermostability.
4. Cavities. Proteins from alicyclobacilli are more closely packed than the analogue molecules in mesophiles.
5. Proline. Thermostable proteins by alicyclobacilli show a higher content of proline and this amino acid is more common at the second position of the β-turns.

This is one last detail on a possible benefit and positive role of *Alicyclobacillus* spp. These microorganisms are generally labeled as spoilers or super-spoilers for acidic drinks; however, Yuan *et al.* [43] heat-inactivated alicyclobacilli cells and used them as adsorbing tools to remove/reduce patulin in apple juice. The removal rate was *ca*. 80% after 24 h.

## 3. Ecology of the Genus *Alicyclobacillus*, with a Special Focus on the Species *A. acidoterrestris*

Spoilage of commercially available pasteurised fruit juice by *Bacillus acidoterrestris* was first reported in Germany in 1982 [44]. Several other cases of spoilage by similar bacteria occurred in Japan, Europe and the U.S.A. in 1990 [45,46]. Though spoilage by *Alicyclobacillus* spp. was previously regarded as sporadic, a 1998 survey by the National Food Processors Association (NEPA) in the USA reported that 35% of the fruit juice manufactures experienced spoilage caused by acidophilic spore-formers suspected to be *A. acidoterrestris* [3,8]. As a matter of fact, *A. acidoterrestris* caused spoilage of isotonic water and lemonade [47], carbonated fruit juice drinks [48], canned diced tomatoes [3] and fruit pulps, Australian shelf-stable iced tea containing berry juice [49], apple, pear, orange, peach, mango and white grape juices [8,30]. *Alicyclobacillus* strains were also isolated from orchard soil and a fruit concentrate production factory in South Africa; many strains were identified as *A. acidoterrestris*, but *A. acidocaldarius* was also recovered [50].

Fruit contaminated by soil during harvest or the use of unwashed or poorly washed raw fruits during processing are the most common sources of *A. acidoterrestris* [8]. Spores are also introduced into the manufacturing facilities by soil associated with employees. Water can also be a source of *A. acidoterrestris* spores; McIntyre *et al.* [51] isolated the same strain of *Alicyclobacillus* from spoiled juice and from water used by a processing facility. Recently, apple and pear flavourings have been reported as significant sources of *A. acidoterrestris* [52].

The fruit juice industry now acknowledges *A. acidoterrestris* as a major quality control target for thermal treatment efficacy [31,32,47,48,53]. It represents the greatest threat of spoilage in acidic foods because spores are able to germinate and grow at low pH [45].

Spoilage by *Alicyclobacillus* is difficult to detect because *A. acidoterrestris* does not produce gas during growth. The spoiled juice appears normal with little or no change in pH. Occasionally, turbidity and/or white sediment may be formed at the bottom of the container. Members of *Alicyclobacillus* genus cause some clarified fruit juices to have a light sediment, cloudiness or haze [3].

However, the most common characteristic of *Alicyclobacillus* contamination is a “smoky”, “medicinal”, “antiseptic” off-odour associated with guaiacol (2-methoxyphenol) [46,48], and other halophenols, including 2,6-dibromophenol and 2,6-dichlorophenol. The odour of the taint has also been described as smoky and pungent [54].

Guaiacol is a product of microbial metabolism in fruit juices and dairy foods. It is formed directly from vanillic acid by nonoxidative decarboxylation [8]. Many soil bacilli can decarboxylate vanillic acid to guaiacol. Vanillic acid is naturally derived from the plant polymer lignin and can be also introduced to the beverage as an ingredient. *Alicyclobacillus* spp. can also convert vanillic acid to vanillyl alcohol, catechol and methoxyhydroquinone [8]. Tyrosine is another possible precursor for guaiacol formation. Apple juice contains approximately 4.1 mg tyrosine/ml juice and orange juice contains 3–13.5 mg tyrosine/ml [27]. Guaiacol production depends on the viable count of alicyclobacilli, strain, storage temperature, oxygen concentration in beverage, use of heat shock which encourages germination of the spores, and, finally, concentration of precursors to guaiacol, such as vanillin and tyrosine in the fruit juice [8,48,55,56].

Fortunately, there is no evidence that *A. acidoterrestris* poses a human health risk. Neither the organism nor its metabolites have been associated with any form of illness and *A. acidoterrestris* is considered a non-pathogen [57]. However, in 2007 an endospore-forming organism was isolated from a blood sample from a 51-year-old woman on blood agar at 37 °C [24]. There is no evidence that this strain was the causal agent of an infection. Based on 16S rRNA gene sequence similarity comparisons, the strain was grouped into the genus *Alicyclobacillus*, most closely related to the type strain of *Alicyclobacillus pohliae* (94.7%), and was named *A. consociatus*. However, a reclassification was proposed for *A. pohliae* and *A. consociatus* as *Effusibacillus pohliae* and *E. consociatus*, respectively, according to phylogenetic and phenotypic analysis showing that the monophyly of the genus *Alicyclobacillus* had been lost [58].

*A. acidoterrestris* in fruit juice does not affect its pH, thus it cannot enhance the growth of other pathogens such as *Clostridium botulinum* [59]. Detection, recovery and identification of *Alicyclobacillus* spp. in juices and other spoiled beverages is a great challenge, because the traditional plate-counting is a time-consuming methods; thus, some researchers have proposed some alternative approaches in the last three years. Table 3 offers a brief overview of the most important advances.

**Table 3:** Overview of the most recent advances for the recovery and identification of *Alicyclobacillus* spp. (2014 and 2015).

Method	Description	Reference
Lipase and esterase fingerprints	Juice incubation at 45 °C for 24 h, cell harvesting and chromatography	[60]
Aptamer-based enrichment 16S rDNA	The method requires a preliminary enrichment step, so it can take up to 1 week. After a mechanical treatment, DNA was quantified through a RT-PCR based approach	[61]
Immunomagnetic separation RT-PCR	Immunomagnetic separation was combined with RT-PCR, by using two probes. The method is highly selective for *A. acidoterrestris*	[38]
FIR	Fourier transformed intra-red spectroscopy (1350–1700/cm), combined with multivariate statistical analysis (Principal Component Analysis and Class Analogy), allows the discrimination between *Bacillus* and *Alicyclobacillus* spp.	[62]
G-quadruplex colorimetric method	*A. acidoterrestris* was grown at 45 °C in presence of vanillic acid; this compound is easily converted to guaiacol and finally to tetraguaiacol (amber-coloured). The reaction is catalysed by G-quadruplex DNA-zyme	[63]
DAS-ELISA	DAS-ELISA (double antibodies sandwich ELISA) assay is based on the two kinds of polyclonal antibodies from Japanese White rabbit. The method shows high sensitivity and excellent agreement with isolation by K medium	[64]

## 4. Alternative Approaches to Mitigate *Alicyclobacillus* Species Associated with Food Spoilage

Pasteurisation treatments on fruit juice are generally used to control bacterial contamination and increase shelf-life. The U.S. Food and Drug Administration requires all fruit juice sold in the United States to be either pasteurised or subjected to an equivalent process to achieve a mandated 5-log pathogen reduction in the juice [65].

Typically fruit juices are pasteurized through a flash treatment, which uses high temperature/short time to preserve the organoleptic and nutritional properties of the juice [66,67]; for example, fruit juice is heated to around 88–96 °C for 30 s to 2 min and then rapidly cooled [68].

Unfortunately, *A. acidoterrestris* spores are able to survive thermal pasteurisation and hot-fill hold processes [9,45,69], but pasteurisation stimulates the germination of the spores. The design of pasteurization processes requires the evaluation of a *P*-value, which is the minimum heat required (time-temperature exposure to heat) to result in a product retaining quality during storage; the following details are required for a robust determination of *P*-value [70]:

- determination of *D*-value and *z*-value of *A. acidoterrestris* spores;
- potential for *A. acidoterrestris* spore growth during product storage for at least 1 month at 25 and 43 °C;
- quality during storage following pasteurization treatments of different severity.

**Figure 1:**
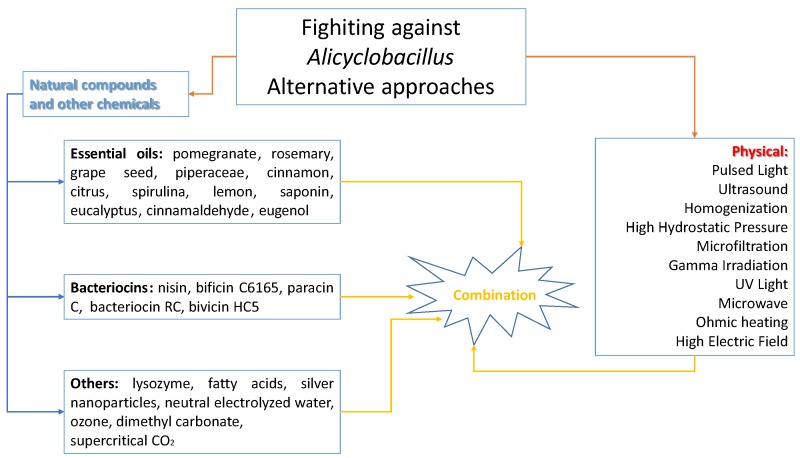
Alternative approaches to control *Alicylobacillus* spp. (overview from 2005 to 2015)

However, the ability of *A. acidoterrestris* spores to survive thermal pasteurisation processes requires the design of alternative processing techniques to pasteurisation [9,69]. An interesting update and overview of the most important alternative approaches to control and/or reduce the contamination by *Alicyclobacillus* spp. is reported in the paper by Tianli *et al.* [71]. Figure 1 proposes a graphical abstract of the most used approaches.

The use of UV light as a germicidal tool is one such promising technology. Advantages associated with UV-C radiation used as a non-thermal method are that no known toxic or significant nontoxic by-products are formed during the treatment, and the treatment requires very little energy when compared to thermal pasteurisation. UV light was used as a suitable means to decrease alicyclobacilli contamination [72,73]; for example, Baysal and Ünlütürk [73] proposed this approach to reduce spore counts on the surface.

Another interesting physical intervention treatment is high-pressure homogenization (HPH). Its efficacy against cells and spores of *A. acidoterrestris* was reported by many authors [74,75]; Bevilacqua *et al.* [74] found that the antimicrobial effect was strain dependent and the spores were less sensible to HPH. The bactericidal activity of HPH could be due to the disruption of the cell wall and outer membrane [76]; pressures could induce a structural rearrangement of proteins, an increased exposure of their hydrophobic regions and the disruption of their supramolecular structure under pressure allowing the components to move freely and become independent of the original structure [76].

A pulsed electric field (PEF) is another non-thermal process, based on high-voltage short pulses delivered to the product placed between two electrodes. Electroporation is believed to be the principle of this method. Application of high-intensity pulsed electric fields destabilizes the microbial cell membrane and causes alterations in ion transport processes, which in turn results in cell damage and death [77]. The efficacy of PEF technology against spore-forming bacteria has been successfully demonstrated in fruit juices with minimal effects on freshness characteristics, such as color, pH value or flavour compounds [78,79].

Ultrasound or ultrasonic waves (US) were defined as electromagnetic waves with frequency beyond 20 kHz. Ultrasound is able to disaggregate bacterial clusters and inactivate bacteria through the mechanical, physical, and chemical effects of acoustic cavitation [80]. Morales-de la Peña *et al.* [79] tested the effectiveness of treatment time and power of US on the inactivation rate of *A. acidoterrestris*. The inactivation mechanism of US might lie in intracellular cavitation, localized heating, cell membranes being thinner, and free radical production [81].

Another interesting approach might be also the use of chemical compounds such us nisin and other bacteriocins that exhibit bactericidal activity against certain bacteria [82], and *A. acidoterrestris* [32]. Nisin is currently the only bacteriocin approved for use in food by the FDA and the World Health Organization [83,84,85]. The bioactivity of nisin is influenced by pH, water activity, content of solids, and the presence of other inhibitory factors [32,82,86,87]. However, some studies assert that the inhibitory action of nisin is lower in clear apple drink than *in vitro* studies, probably due to the ability of this compound to bind to some apple particles, although nisin would also be absorbed onto some particles in orange or mixed fruit drinks [82]. Nevertheless, nisin is heat stable and the beneficial effects of its inclusion prior to pasteurisation would be twofold: it would enhance the effect of the heat process, and residual nisin would prevent outgrowth of surviving spores.

In addition, some authors recommended the use of lysozyme for its bactericidal effectiveness, although the effect was strain-dependent [88].

Not least is the application of essential oils (EOs) against alicyclobacilli. The use of essential oils could be considered a new approach, as the stabilization of juices could be achieved through some natural molecules extracted from plants and fruits. Therefore, the consumer would not consider these antimicrobials as chemicals, but rather as natural ingredients of juices that might be added to commercial juices to improve the flavour of the products.

Cinnamaldeyde (100–500 ppm) was able to prevent spore germination of *A. acidoterrestris* for at least 13 days [89]; otherwise, eugenol acted as a strengthening element and, combined with cinnamaldeyde, reduced its amount in the system. Specifically, the experiments were performed in a commercial apple juice, thus highlighting that spore germination could be inhibited through the use of 80 ppm of eugenol and 40 ppm of cinnamaldehyde or alternatively through the combination of 40 ppm of eugenol with 20 ppm of cinnamaldehyde [90].

Although many authors have previously reported the suitability of citrus extracts as natural preservatives for the inhibition of a wide range of microorganisms [91,92], to the best of our knowledge there are few data on the spores of *A. acidoterrestris*. The results of *in vitro* assay [93] confirmed that the bioactivity of citrus extracts was related to their concentrations with an effect called the “dose dependence effect” (DDE). Specifically, citrus and lemon extract showed MIC values (minimal inhibitory concentration) from 160 to 500 ppm against *A. acidoterrestris* spores*.*

Eucalyptus extracts and three compounds from *Eucalyptus maculata* were tested against spoiling microorganisms, and the effect on *A. acidoterrestris* was significant [94].

## 5. Conclusions

Cases of spoilage by *Alicyclobacillus* spp. of pasteurised fruit juice products have increased considerably in the last few years [31,47,48,53,89]. At present, the source of fruit juice contamination remains unclear. However, as members of the genus *Alicyclobacillus* are soil-borne organisms, it is thought that contaminated fresh fruit introduced during processing without proper cleaning leads to contamination and subsequent spoilage [45,69,95].

Standard pasteurization processes utilizing temperatures of 85 and 95 °C, are commonly used to destroy pathogens such as *Escherichia coli* O157:H7 and *Salmonella* and are not effective against thermotolerant spore-forming spoilage bacteria [45]. Thermal processes able to affect *Alicyclobacillus* spores are not feasible as they are potentially harmful to product quality [3,96].

The ability of *A. acidoterrestris* spores to survive thermal pasteurisation and hotfill and hold processes used during fruit processing and fruit juice production requires the design of alternative techniques to reduce bacterial contamination [9,45,69]. The use of non-conventional approaches to control alicyclobacilli could be considered a promising method for the juice industry; however, literature data refer to laboratory media and to experiments performed at the lab scale. A future strategy would be the scaling up of lab techniques to the industry level, in order to pinpoint whether the designed approaches could be applied successfully in a real system.
